# A retrospective study on perineal lacerations in vaginal delivery and the individual performance of experienced mifwives 

**DOI:** 10.1186/s12884-015-0703-0

**Published:** 2015-10-22

**Authors:** Johannes Ott, Evelyn Gritsch, Sophie Pils, Sophie Kratschmar, Regina Promberger, Rudolf Seemann, Sabine Fürst, Dagmar Bancher-Todesca, Christa Hauser-Auzinger

**Affiliations:** Department of Obstetrics and Fetomaternal Medicine, Medical University of Vienna, Waehringer Guertel 18-20, 1090 Vienna, Austria; Midwifery Services, General Hospital of Vienna, Waehringer Guertel 18-20, 1090 Vienna, Austria; Department of Gynecology, St. John of God Hospital Vienna, Johannes von Gott Platz 1, 1020 Vienna, Austria; Department of for Cranio- and Maxillofacial Surgery, Medical University of Vienna, Waehringer Guertel 18-20, 1090 Vienna, Austria

**Keywords:** Vaginal delivery, Perineal trauma, Complication, Quality management, Midwife

## Abstract

**Background:**

Medical staff’s influence on patient outcomes has become a subject of interest. We evaluated experienced midwives and compared their performance concerning perineal lacerations (PL).

**Methods:**

In a retrospective cohort study, 1937 women with singleton pregnancies who had delivered spontaneously with a cephalic presentation by experienced midwives in the Medical University of Vienna from January 2009 to April 2014 were included. As predictive parameters, we included basic patient-, pregnancy- and delivery-related characteristics including the individual midwife who delivered the child. The incidence of PL was the main outcome measure.

**Results:**

Overall PL and severe PL were found in 508/1937 (26.2 %) and 19/1937 women (1.0 %), respectively. In a multivariate analysis for PL of any degree, maternal age (ß = 0.170 ± 0.080), gestational age at delivery (ß = 0.190 ± 0.320), and birth weight (ß = 0.002 ± 0.000) significantly increased the risk, whereas multiparity (ß = −0.379 ± 0.141) and mediolateral episiotomy (ß = −1.514 ± 0.284) decreased it (*p* < 0.05). In addition, the individual midwife who delivered the child was a significant influencing factor, with ß-values ranging from −0.028 to 0.899 compared to the reference midwife. For severe PL, the midwife was not of significant influence.

**Conclusions:**

The individual midwife is an independent factor that influences the risk for overall PL, not for severe PL. Other risk factors include maternal age, gestational age at delivery, birth weight, parity and episiotomy.

## Background

Quality control and continuous quality improvement are developing fields, and have become important aspects of modern medicine within the last few decades [1]. In recent years, the influence of the medical staff on patient outcomes has gained interest, especially in the field of surgery [2]. Accordingly, every individual surgeon would have an individual risk profile for surgical outcome [3, 4]. Notably, this applies not only to trainees, but also to experts, as demonstrated in the field of thyroid surgery [1, 3, 4]. This clearly suggests that these variations at the individual level go beyond surgical volume. As already discussed, this might be due to variations in manual dexterity, but also to an individual’s ability to assimilate refined techniques [3].

These interesting data suggest that the staff’s individual performance might also be of a major influence in other medical fields. In the field of obstetrics, many healthcare organizations and commission advise regular interdisciplinary training including both midwives and doctors, at least for emergencies. This should overcome the most common errors, namely confusion in roles and responsibilities, poor communication between staff as well as errors on the individual level including failure to prioritize management actions and failure to perform clinical tasks in a structured manner [5]. The fact that the individuals’ performances is of impact is supported by the beneficial effect of simulation teaching in the learning of midwifery [6].

In obstetrics, one important complication is the occurrence of perineal lacerations in the course of a vaginal delivery. Perineal lacerations occur in up to two-thirds of women and have been claimed to cause social problems and affect a mother’s psychological well-being [7]. The classification of perineal lacerations is well-known: first or second degree lacerations include the skin, subcutaneous and muscle layers, but exclude the anal sphincter. The third- or fourth degree category (severe perineal lacerations) include partial or complete tears to the anal sphincter or the rectum, respectively [7].

Risk factors for severe perineal lacerations include increasing birth weight, operative vaginal deliveries, labor induction and augmentation [8]. Moreover, manual protection of the perineum could not be proven to be beneficial according to meta-analyses of randomized trials [9]. It has, however, been argued that randomized trials only investigated separate, specific interventions, and, thus, might not be appropriate for the evaluation of the highly complex process of delivery, which integrates several different maneuvers [10, 11]. This includes communication between the midwife/obstetrician and the delivering woman, perineal support, and a delivery position that would allow visualization of the perineum during the last few minutes of delivery, among other factors [12]. It seems plausible that this process likely depends on the individual person who cares for the delivering woman, and, thus, it was our study objective to quantify this possible contributing factor. With regard to quality control, this seems of great importance to us. If differences in obstetric performance at an individual level would have a major impact on outcome, those with a poorer performance could be identified and re-training could be offered.

In Austria, midwives usually perform spontaneous deliveries. Thus, we retrospectively evaluated all experienced midwives in our department and compared their performance concerning perineal lacerations as the major outcome parameter.

## Methods

### Patient population

From January 2009 to April 2014, 5754 women had delivered vaginally at our department. For our retrospective cohort study, we applied the following inclusion criteria to select our patient population: women who were delivered at or after 24 weeks of gestation by experienced midwives who had performed >300 deliveries before the study period, and who had delivered a minimum of 100 women within the study period. The latter criterion was chosen in order to guarantee performance balanced data set. Exclusion criteria were as follows: women with non-cephalic presentations; multiple pregnancies and intrauterine fetal death; instrumental deliveries, since in these cases, both the midwife and the obstetrician exert maneuvers that can influence perineal integrity; and women in whom the final stage of delivery had been assisted by more than one midwife for training purposes, and, thus, the exact roles (leading role) in this setting could not be clearly assigned to one of them. In our study population, there was no case of shoulder dystocia. Application of these stringent criteria resulted in a total population of 1937 women.

### Study design and data collection

Data acquisition was performed by retrospective chart review. In our department, the PIA Fetal Database software (GE-Viewpoint, Wessling, Germany) is used as the basic perinatologic database. The major outcome parameter was the incidence of perineal lacerations. A second analysis focused on severe perineal lacerations only (i.e., of third- and fourth-degree). At our department, the obstetrician in charge and the midwife assess the integrity of the perineum together for all women following a vaginal birth.

During the study period, the procedures and manoeuvers were not standardized in our hospital for the second stage of labor. Thus, it was up to the midwife to choose the final position for delivery together with the delivering woman. It was also up to the midwife whether to use perineal support. However, delivery was always accomplished with uterine contractions (and not between uterine contractions).

As predictive parameters, we included a woman’s age; the woman’s ethnicity (Caucasian, Asian, Turkish or other); the completed week of gestation at delivery; parity; the use of epidural analgesia; the use of a episiotomy that was mediolateral in all cases; the duration of the second stage of delivery; birth weight; head circumference; posterior orientation; the woman’s final position for delivery subdivided into lithotomy, side lying, sitting, all-four, and standing position, as well as water deliveries; and the individual midwife (*n* = 14) who delivered the child. The midwife who had delivered the most children before the study period was chosen as the reference midwife (i.e., midwife number 1).

The study was approved by the local ethics committee of the Medical University of Vienna (IRB number 2128/2013). Thereby, permission to access the database containing medical records used was granted. Written informed consent could not be obtained due to the retrospective study design.

### Statistical analysis

Concerning the statistical power, an “a priori” sample size calculation was performed for the z-test of a two-tailed logistic regression model with an effect size of 0.3 (according to an estimated rate of perineal lacerations of 30 %). In order to prove a two-fold risk for at least one individual midwife (1/14, 7.1 %) evaluated as a fixed factor with a power of 95 % and an alpha of 5 %, a total sample size of 1746 was calculated to be sufficient.

Nominal variables are reported as numbers and frequencies, and continuous variables with median and range. The probabilities of perineal lacerations were estimated in generalized linear mixed models with the logit link function, i.e., logistic regression models. Univariate analyses were followed by a multivariate analysis of significant factors. Coefficient estimates β and standard error se(β), and corresponding p-values are given for these analyses. Statistical analysis was performed using the SPSS 17.0 software (SPSS Inc., 1989–2009). Differences were considered statistically significant if *p < 0.05*.

## Results

Perineal lacerations of any grade were found in 508/1937 women (26.2 %), with an incidence of 1.0 % of severe perineal lacerations (19/1937). Table 1 lists basic patient characteristics and other possible risk factors for perineal lacerations of any grade, including age, ethnicity, gestational age at delivery, parity, use of epidural anesthesia, duration of the second stage of labor, mediolateral episiotomy, the final position for delivery, birth weight, the child’s head circumference as well as the midwife who delivered the child. It also provides an overview of statistical predictive models. In short, in univariate analysis, maternal age, gestational age at delivery, birth weight, and head circumference were positively correlated with the incidence of perineal lacerations, whereas women who had given birth to ≥2 children previously and/or who had undergone mediolateral episiotomy experienced a decreased risk. In addition, the individual midwife who delivered the child was a significant influencing factor. After multivariate analysis, all of these factors, apart from the child’s head circumference, remained significantly predictive. As is evident from Table 1, after correction for all influencing factors, there were only two midwives (numbers 2 and 12) with a decreased risk compared to the reference midwife number 1, as indicated by the negative ß-value.

**Table 1:** *Predictive factors for perineal lacerations (grades 1–4)* and results of the univariate and multivariate analysis

				Univariate analysis			Multivariate analysis		
		Perineal laceration (*n* = 508)	No perineal laceration (*n* = 1429)	ß	SE (ß)	*p*	ß	SE (ß)	*p*
Age (years)		30.6 (25.8;35.0)	29.9 (25.1;34.2)	0.170	0.080	*0.033*	0.026	0.009	*0.004*
Ethnicity	Caucasian	401 (78.9)	1112 (77.8)	reference		0.358	-	-	-
	Asian	19 (3.7)	39 (2.7)	0.301	0.286		-	-	
	Turkish	62 (12.2)	211 (14.8)	−0.205	0.156		-	-	
	Other	26 (5.1)	67 (4.7)	0.073	0.238		-	-	
Gestational age at delivery (weeks)		40 (39;40)	39 (38;40)	0.190	0.320	*<0.001*	0.084	0.052	*0.045*
Parity	0	200 (39.4)	594 (41.6)	reference		*<0.001*	reference		*<0.001*
	1	214 (42.1)	427 (29.9)	0.398	0.117		0.072	0.127	
	≥2	94 (18.5)	408 (28.6)	−0.379	0.141		−0.867	0.156	
Epidural analgesia		120 (23.6)	290 (20.3)	0.195	0.123	0.115	-	-	-
Duration of the 2^nd^ stage of labor (min)		33 (17;65)	30 (15;55)	0.002	0.002	0.353	-	-	-
Mediolateral episiotomy		14 (2.8)	163 (11.4)	−1.514	0.284	*<0.001*	−1.776	0.291	*<0.001*
Posterior orientation		6 (1.2)	12 (8.0)	0.345	0.503	0.493	-	-	-
Final position for de-livery	Lithiotomy	459 (90.4)	1292 (90.4)	reference		0.527	-	-	-
	Side lying	23 (4.5)	83 (5.8)	−0.248	0.242		-	-	
	Sitting	10 (2.0)	16 (1.1)	0.565	0.407		-	-	
	All-four	9 (1.8)	18 (1.4)	0.342	0.412		-	-	
	Standing	3 (0.6)	6 (0.4)	0.342	0.709		-	-	
	Water del.	4 (0.8)	14 (1.0)	−0.218	0.570		-	-	
Birth weight (g)		3470 (3160;3790)	3290 (2980;3620)	0.002	0.000	*<0.001*	0.001	0.000	*<0.001*
Head circumference (cm)		35 (34;36)	34 (33;35)	0.173	0.030	*<0.001*	0.060	0.046	0.187
Midwife who delivered the child	1	19 (3.7)	92 (6.4)	reference		*0.004*	reference		*0.036*
	2	22 (4.3)	95 (6.6)	0.115	0.346		−0.028	0.357	
	3	53 (10.4)	117 (8.2)	0.785	0.302		0.645	0.315	
	4	23 (4.5)	80 (5.6)	0.331	0.346		0.188	0.361	
	5	44 (8.7)	101 (7.1)	0.746	0.310		0.526	0.323	
	6	44 (8.7)	128 (9.0)	0.510	0.307		0.365	0.319	
	7	55 (10.8)	93 (6.5)	1.052	0.304		0.899	0.317	
	8	34 (6.7)	116 (8.1)	0.350	0.319		0.316	0.332	
	9	52 (10.2)	108 (7.6)	0.846	0.303		0.598	0.316	
	10	30 (5.9)	100 (7.0)	0.373	0.327		0.225	0.340	
	11	31 (6.1)	81 (5.7)	0.617	0.329		0.638	0.346	
	12	31 (6.1)	129 (9.0)	0.152	0.322		−0.014	0.333	
	13	33 (6.5)	101 (7.1)	0.459	0.322		0.448	0.335	
	14	37 (7.3)	88 (6.2)	0.711	0.319		0.473	0.332	

Data are reported as median (interquartile range) for numerical parameters or numbers (frequency) for categorical parameters; italic letters indicate statistical significance

For severe perineal laceration rates, the individual incidences per midwife ranged from 0 (midwives number 4, 10, and 14) to 2.0 % (midwife number 7). However, these differences were not significant in univariate analysis (*p = 0.996*). We, thus, refrained from further analyses of predictive factors for severe perineal lacerations, since evaluation of “canonical” risk factors was not the objective of the present study. Notably, 47.4 % (9/19) of the affected women were multiparous with two of them having given birth to two or more children previously (10.5 %). None of the women had experienced a previous severe perineal laceration.

## Discussion

The major finding of this retrospective study is that the midwife is an independent predictive factor for the occurrence of perineal lacerations during a vaginal delivery. The data are somehow in line with the observation of highly varying rates of severe perineal lacerations in midwife-conducted deliveries between different centers [13]. However, concerning severe lacerations, our study did not reveal differences between the individual midwives which might be due to the small sample size. A much larger population would be needed to test a complication with an incidence ranging from 0-2 % per individual midwife.

It could be argued that before addressing quality issues on an individual level, one should compare outcome data on an institutional level. Our rate of overall perineal lacerations (26.2 %) is comparable to published data. A recent multicenter study revealed a rate of 21 % [14]. In an older report an even higher rate of up to two thirds was found [7]. The incidence of severe lacerations of 1.0 % found in our data set is comparable to the results of published data ranging from about 1.0 % [7, 13], to about 4.5 according to the birth center and the risk profile [13, 14]. Moreover, other risk factors for perineal lacerations in our report are in line with the published literature [8, 11, 14]. These include gestational age, parity, and birth weight. It should be emphasized that we evaluated perineal lacerations of all grades, and, thus, a mediolateral episiotomy was protective in our analyses, which is contrary to risk factor analyses for severe lacerations only [8]. In contrast to previous studies that have been reviewed by Pergialiotis et al. [8], the woman’s ethnicity was not predictive which might be caused by the fact that we analyzed all grades of perineal lacerations instead of severe cases only and by the small number of ethnicities other than Caucasian. Moreover, the position of delivery was not of influence in our analysis, which is likely due to the small number of positions other than lithotomy. In order to increase the reliability of our data, we excluded all instrumental deliveries, since not only the midwife, but also the obstetrician, likely exerts maneuvers that could influence perineal integrity in these cases. Thus, we consider it sound to state that the midwife herself was responsible for all manual actions during delivery in the presented cohort study. Moreover, the obstetrician was responsible for inspection of the perineum and, thus, the major outcome parameter should be objective.

All in all, we consider the requirement of benchmarking our results against other published data fulfilled to look at the individual midwife. Since several other parameters were independent risk factors for perineal lacerations in addition to the individual midwife in the multivariate model, we can rule out that some of the midwives cared for more complex deliveries. Thus, differences in handling, probably also in communication with the delivering woman, likely contributed to the differences in outcomes. In our department, midwives usually deliver children based on general recommendations about the last stage of delivery. This includes that the hand of the midwife should control the speed of the head crowning through the vaginal introitus, while the other hand should support the perineum. The mother would then be asked to stop pushing and, when most of the head is out, the perineal ring would be pushed under the neonate’s chin as described previously [12]. It, however, must be noted that literature comparing this “hands-on” method to the “hands-off” or the “hands-poised” methods is controversial. Several studies showed favorable results for the latter two methods [15–17], others concluded that a clear beneficial effect could be seen for the “hands-on” method [12, 18]. These observations are in line with the last Cochrane database systematic review on this topic stating that “hands-off” versus “hands-on” showed no effect on third- and fourth-degree tears [5] as well as with a recent meta-analysis [19]. Accordingly, current midwifery guidelines recommend that either approach is appropriate [20]. However, randomized controlled trials evaluating different techniques of perineal support may not be the only approach for investigating such complex interventions. Taken these considerations together, it is likely that other modifiable factors contributed to the differences in individual outcomes. Other recommended strategies include emphasis on selective mediolateral episiotomy, good visualization of the perineum at birth and communication with the mother regarding slow pushing [12]. Unfortunately, we could not evaluate the latter two factors due to the retrospective study design. However, in this context, it must be emphasized once more that all analyzed midwives were experienced and had performed several hundred vaginal deliveries before the study period. Moreover, from our experience, the extent to which these maneuvers are exerted differs between the midwives. This is in line with the previously published observations that many midwives preferred a personalized approach rather than an a standardized recommended technique and that many midwives will respond to different clinical scenarios by changing technique [21, 22].

To compare the performance of individuals is not only of interest in order to detect those with a weaker performance and offer them training, but also to identify those with highly favorable outcomes and analyze their method of handling deliveries. Thus, new strategies could be developed that could be tested in subsequent studies. One should learn from the best and this is of a considerably high relevance, especially in such a complex field as a vaginal delivery where many circumstances likely are associated with overall outcome, perineal lacerations among them.

Of course, this study cannot evaluate the midwives’ willingness to revise handling strategies and learn from colleagues. As reported previously for general surgeons, this willingness can be low, and, thus, new strategies might not be adapted, especially among those who consider themselves experts [3]. Whether confrontation of the midwives with their personal rates of perineal lacerations would lead to improvements in outcome shall be the aim of future studies. It is obvious that one would have to handle such an intervention with care and that one individual can receive information only about her own performance with the others’ results remaining blinded.

The retrospective design of the study naturally is a limitation, since several possible influencing factors had not been documented in the electronic system routinely and, thus, could not be evaluated. These include perineal support. However, it was up to the midwife whether to use perineal support or not. The study was based on the assumption of variations in handling strategy on an individual level. Hence, the lack of exact information on perineal support does not decrease the relevance of our major finding, namely that the midwife was an independent predictive factor. One could also be concerned whether perineal lacerations were diagnosed correctly. It has been claimed that rates of perineal lacerations were associated with clinical ascertainment [23] and that the level of recognition could vary [24, 25]. Notably, in a recent Australian report, only 71 % of the midwives reported that they had received training in diagnosing severe perineal lacerations and only 16 % of these felt highly confident in this diagnosis [22]. However, as mentioned in the Methods section, both the midwife and an obstetrician usually assess the integrity of the perineum together for all women which should minimize diagnostic bias. Moreover, one might discuss the clinical relevance of low-grade perineal lacerations. However, these can also cause pain and can even lead to wound-healing problems in rare cases. Nevertheless, our data suggest that some cases of lacerations might be avoided by more careful handling, and, without a doubt, for the mother, the optimal delivery would, of course, be one without any complications at all. We, thus, consider the topic of perineal lacerations of relevance, regardless of the severity.

## Conclusions

The individual midwife is an independent factor that influences the risk for perineal lacerations. Future studies are warranted to prove our results and to further explore the optimal way of handling during a vaginal delivery. Moreover, we hope to introduce the topic of a staff-based approach to patient outcomes in the field of obstetrics and fetomaternal medicine.

## Ethic, content and permissions

The study was approved by the Institutional Review Board of the Medical University of Vienna (IRB number 2128/2013). Due to the retrospective study design, patients did not sign a written informed consent.

## Consent to publish

Not applicable.
